# Whole Genome Sequence Analysis Suggests Intratumoral Heterogeneity in Dissemination of Breast Cancer to Lymph Nodes

**DOI:** 10.1371/journal.pone.0115346

**Published:** 2014-12-29

**Authors:** Kevin Blighe, Laura Kenny, Naina Patel, David S. Guttery, Karen Page, Julian H. Gronau, Cyrus Golshani, Justin Stebbing, R. Charles Coombes, Jacqueline A. Shaw

**Affiliations:** 1 Department of Cancer Studies and Molecular Medicine, Robert Kilpatrick Clinical Sciences Building, Leicester Royal Infirmary, Leicester, LE2 7LX, United Kingdom; 2 Division of Cancer, Imperial College, Hammersmith Hospital Campus, London, W12 0NN, United Kingdom; Shanghai Jiao Tong University School of Medicine, China

## Abstract

**Background:**

Intratumoral heterogeneity may help drive resistance to targeted therapies in cancer. In breast cancer, the presence of nodal metastases is a key indicator of poorer overall survival. The aim of this study was to identify somatic genetic alterations in early dissemination of breast cancer by whole genome next generation sequencing (NGS) of a primary breast tumor, a matched locally-involved axillary lymph node and healthy normal DNA from blood.

**Methods:**

Whole genome NGS was performed on 12 µg (range 11.1–13.3 µg) of DNA isolated from fresh-frozen primary breast tumor, axillary lymph node and peripheral blood following the DNA nanoball sequencing protocol. Single nucleotide variants, insertions, deletions, and substitutions were identified through a bioinformatic pipeline and compared to CIN25, a key set of genes associated with tumor metastasis.

**Results:**

Whole genome sequencing revealed overlapping variants between the tumor and node, but also variants that were unique to each. Novel mutations unique to the node included those found in two CIN25 targets, *TGIF2* and *CCNB2*, which are related to transcription cyclin activity and chromosomal stability, respectively, and a unique frameshift in *PDS5B*, which is required for accurate sister chromatid segregation during cell division. We also identified dominant clonal variants that progressed from tumor to node, including SNVs in *TP53* and *ARAP3*, which mediates rearrangements to the cytoskeleton and cell shape, and an insertion in *TOP2A*, the expression of which is significantly associated with tumor proliferation and can segregate breast cancers by outcome.

**Conclusion:**

This case study provides preliminary evidence that primary tumor and early nodal metastasis have largely overlapping somatic genetic alterations. There were very few mutations unique to the involved node. However, significant conclusions regarding early dissemination needs analysis of a larger number of patient samples.

## Introduction

The presence of tumor spread to local lymph nodes is one of the most important prognostic factors affecting patient survival in breast cancer [1]–[4]. Many treatment strategies are largely based on protein expression measurements of steroid hormone receptors and Her2, which broadly segregates tumors into 5 molecular subtypes [5]. However, genetic profiling of primary tumors suggests that the landscape is much more complex than this, with the identification of at least 10 distinct subtypes by the METABRIC consortium [6], which has implications for both prognosis and treatment [5], [7].

In the era of targeted therapeutics, intratumoral heterogeneity is being increasingly recognized as an important barrier to the success of cancer treatments. Multiregion sequencing of samples taken from the same renal cell carcinoma and distant metastases revealed that more than 60% of all somatic mutations were not detectable across every tumor biopsy that was taken, suggesting that we have previously underestimated the clinical impact of genetic complexity in individuals as a result of heterogeneity [8]. Indeed, the intratumoral heterogeneity seen in renal carcinoma led to phenotypic diversity in the form of activating mutations in *MTOR*, which may predict for intrinsic resistance to drugs targeting the PI3K-MTOR pathway. On the other hand, intertumoral heterogeneity has been equally well described previously for primary breast cancer [9], and even in the phenotypically diverse but rare metaplastic breast cancer subtype [10].

The origin of tumor heterogeneity is frequently debated and it is believed that it could arise as a consequence of clonal evolution [11], [12]. Meanwhile, chromosomal instability (CIN) is a hallmark of human cancer that is characterized by elevated rates of chromosome miss-segregation [13], [14] and is thought to be due to specific gene alterations that arise before malignant transformation occurs. Chromosomal instability can give rise to a heterogeneously aneuploid tumor that could enable selective adaptation and evolution; moreover, CIN is a process that is required for metastasis and resistance to therapy to occur [15], [16]. Identifying genetic drivers of CIN is thus central to further understanding this type of genomic instability. and —in this way— understanding the origin of tumor heterogeneity.

In this study, we sought to define genetic variability early in the metastatic process through the comparison of a primary breast tumor with paired locally-involved axillary lymph node in DNA isolated from the same patient by whole genome sequencing.

## Materials and Methods

Tissue samples were provided by the Imperial College Healthcare NHS Trust Tissue Bank. Other investigators may have received samples from these same tissues. We performed whole genome sequencing of DNA from a homogenized primary breast tumor, locally-involved axillary lymph node, and normal tissue (whole blood) from a patient who had no clinical evidence of visceral metastases. Following patient consent, a fresh tumor and lymph node sample were each snap-frozen from the resected specimen. The specimen was obtained at the time of mastectomy and axillary node clearance for a 10 cm, grade 2, invasive ductal carcinoma - all (22/22) lymph nodes were involved. Staging investigations did not reveal any evidence of distant metastases. The project was approved by the Imperial College Healthcare NHS Trust tissue bank in accordance with the Human Tissue Act (HTA) guidelines. Tumor and node were microdissected to ensure 90% quality of neoplastic cells and verified by an experienced histopathologist. There had been no previous anticancer treatment.

DNA was extracted using the Gentra Puregene Cell Kit (QIAGEN). Whole genome sequencing of samples was carried out by Complete Genomics Inc.. Sequencing involved the use of a four adaptor library protocol, as detailed in Drmanac [17]. Briefly, sequencing substrates were generated by means of genomic DNA fragmentation and recursive cutting with type IIs restriction enzymes and directional adaptor insertion. The resulting circles were then replicated with φ29 polymerase and rolling circle replication (RCR) [18] by synchronized synthesis to obtain hundreds of tandem copies of the sequencing substrate, referred to as DNA ‘nanoballs’ (DNBs), which were adsorbed to silicon substrates with grid-patterned arrays to produce DNA nanoarrays. High accuracy cPAL sequencing chemistry was then used on automated sequencing machines to independently read up to 10 bases left and right of each of the four adaptor insertion sites (i.e., a total of 8 oligonucleotide anchor insertion sites), resulting in a total of 31- to 35-base mate-paired reads (62 to 70 bases per DNB).

DNA nanoball intensity information proceeded with the following steps: 1, background correction; 2, image registration; and 3, intensity extraction, during which the intensity data from each field was subjected to base calling, which itself involved four major steps: 1, crosstalk correction; 2, normalization; 3, elucidation of the base present; and 4, raw base score computation. The resulting mate-paired reads were aligned to the hg19/NCBI Build 37 reference genome in a two-stage process: first, left and right mate-pairs were aligned independently using indexing of the reference genome; second, for every location of a single arm identified in the first stage, local alignment at approximately the mate-pair distance was applied to the other arm. At locations selected for likely differences from reference, mapped reads were assembled into a best-fit, diploid sequence with a custom software suite employing both Bayesian and de Bruijn graph techniques as described previously [19]. This process yielded diploid reference with either variant or no-calls at each genomic location, and with associated variant quality scores.

Variants were called using CGA Tools (Complete Genomics Inc.). For the purposes of this study, ‘variants’ includes single nucleotide variants (SNVs), insertions, deletions, and substitutions. In addition, we judged variants as ‘known’ if they were already listed in dbSNP v132 [20], whilst we also overlapped each called variant with COSMIC [21]. Gene enrichment was performed with the Genetic Association Database (GAD) [22] and the Gene Ontology (GO) [23]. In all of our analyses, we narrowed our focus to variants called in genes and their flanking regions and used only those that had passed CGA Tools quality control.

## Results and Discussion

**Figure 1:**
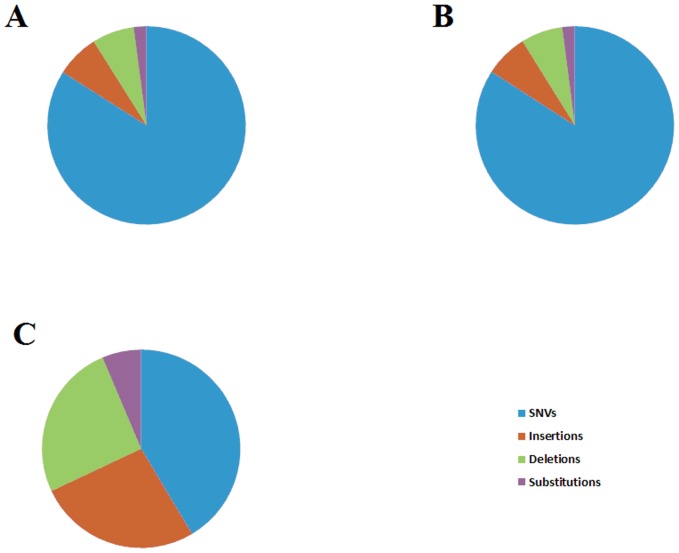
Changing proportion of SNVs, insertions, deletions, and substitutions across samples. Variants that passed QC and that were called at any read-depth in genes and gene-coding regions were selected. The proportions of these variant types changed when looking at those unique to the node, with much higher proportions of insertions, deletions, and substitutions being found. A, Tumor; B, Node; C, unique to node.

The output for each sample exceeded 380,000 gigabase (Gb) (mean 382,072 Gb), with >97% of the genome of each sample being mapped successfully to the reference genome. Depth of coverage over all mapped bases at 40x or higher was >92% and >95% when considering the exome (Table 1). There were more SNVs called than other variant. The proportion of SNVs, insertions, deletions, and substitutions was similar between matched tumor and node (means of 84.1%, 7%, 6.9%, and 2%, respectively) (Fig. 1); however, the distribution of variants that were unique to the node differed, with a higher percentage of insertions (26.6%) and deletions (25.6%) - there was also a modest increase (6.4%) in substitutions. This may reflect increased genomic instability, which has been reported previously in breast cancer [24], [25], or structural CIN (sCIN), a potential hallmark of metastatic cancer [26]. Variants unique to the node overlapped a total of 347 genes and GO enrichment of these genes revealed three significant terms (*P*<0.0001): keratinocyte differentiation (GO:0030216); keratinization (GO:0031424); and epidermal cell differentiation (GO:0009913). Of these 347 genes, 55 had a variant that resulted in a frameshift in the coding sequence, and GO enrichment of this sub-group revealed no significant term (using *P* = 0.01 as cut-off). When we focused on those variants likely to produce a functional impact (i.e., splice acceptor/donor variants, missense, and also insertions, deletions, or substitutions in coding regions) a total of 4,763 genes contained a variant or variants of likely functional impact in the tumor and 4,739 in the node. The top GAD term associated with these was breast cancer (*P*<0.001 for tumor and node), whilst the top-associated GO biological process was cell surface receptor linked signal transduction (*P*<0.0001 for both tumor and node). Considering genes whose variants were unique to the node, the top GO biological process term was regulation of transcription (DNA-dependent) (*P*<0.01), which suggests that transcriptional changes promote metastasis. We observed many variants located upstream of the transcription start-site (TSS), within the promoter region. A variant in this region could potentially alter transcription of the gene downstream of the variant.

**Table 1:** Total output (Gb) and depth of coverage for each sample.

			Depth of coverage									
			Whole genome (%)					Exome (%)				
Sample	Output (Gb)	Successfully mapped (%)	≥5x	≥10x	≥20x	≥30x	≥40x	≥5x	≥10x	≥20x	≥30x	≥40x
**Normal blood**	392,946	97.7	99.5	99.1	98.0	96.2	93.6	99.9	99.7	99.3	98.6	97.3
**Primary tumor**	384,178	97.4	99.5	99.1	97.6	95.4	92.2	99.8	99.7	99.1	97.9	96.1
**Axillary lymph-node**	381,091	97.4	99.5	99.0	97.5	95.3	92.2	99.8	99.6	99.0	97.8	95.9

The amount that was successfully mapped to the reference genome for each sample was >97%, with a mean of 92.7% of each base achieving ≥40x coverage (or 96.4% for the exome fraction).

There were 6 tumor and/or node mutations listed in the COSMIC database that were not present in the matched normal blood sample: three were unique to the tumor; the node harbored a single unique mutation; and two mutations were common to both (Table 2). The unique mutation in the node was an insertion, leading to a frameshift in the coding sequence of *PDS5B*, a gene that interacts with the cohesion complex to maintain accurate sister chromatid segregation during mitosis and meiosis and suggested previously as a tumor suppressor [27], [28]. Of note, frameshifts in *PDS5B* have been reported recently in gastric and colorectal carcinomas with high microsatellite instability [28]. The two mutations common to the primary tumor and node were SNVs in *ARAP3* and *TP53*. *TP53* is a tumor suppressor which functions as a transcription factor and also plays a key role in the cellular response to stress [29]. Germline mutations in *TP53* causes Li-Fraumeni Syndrome [30] and somatic mutations are found in many human cancers [31]. ARAP3, mediates rearrangements to the cytoskeleton and cell shape; in a study by Yagi [32], the expression and phosphorylation of ARAP3 was found to reduce invasiveness of gastric carcinoma to the peritoneum, a function that was suppressed by mutations within the *ARAP3* gene. The mutations unique to the primary tumor may have been derived from a sub-clone unrelated to the metastasis. These included SNVs in *MUC12* and *ZNF99*, two largely unresearched genes, and a single base deletion in *FHOD1*, a gene found to participate in cytoskeletal changes during endothelial-mesenchymal transition (EMT) but whose depletion reduced the ability of EMT cancer cells to progress *in vivo* [33]. In our study, it is possible that the single base deletion in *FHOD1* reduced the activity of the gene and, in turn, reduced the metastatic potential of the sub-clone in which the deletion appeared, and might explain why we failed to find this SNV in the nodal metastasis. The similarities and differences between tumor and involved node may indicate intratumoral heterogeneity, that the nodal metastasis was derived from a minor sub-clone of the tumor *not* represented in the tumor tissue that was sequenced or may reflect sampling when the tissue was selected for sequencing.

**Table 2:** COSMIC mutations called in the primary tumor and axillary lymph node.

Chromosome	Start bp	End bp	Type	Reference	Variant	COSMIC ID	Symbol	Tumor?	Node?
5	141033869	141033870	SNV	T	G	COSM32578	*ARAP3*	Yes	Yes
7	100612086	100612087	SNV	A	G	COSM147730	*MUC12*	Yes	No
13	33344887	33344887	Insertion	-	A	COSM85618	*PDS5B*	No	Yes
16	67267851	67267852	Deletion	G	-	COSM50200	*FHOD1*	Yes	No
17	7577093	7577094	SNV	G	A	COSM10704	*TP53*	Yes	Yes
19	22954575	22954576	SNV	A	G	COSM140394	*ZNF99*	Yes	No

Mutations were not present in the normal blood sample. Three mutations were unique to the tumor whilst the node harbored a single unique mutation: a frameshift in the coding sequence of *PDS5B*, a gene that interacts with the cohesion complex to maintain accurate sister chromatid segregation during mitosis and meiosis and suggested previously as a tumor suppressor [27], [28].

In order to more accurately detect variants indicative of ‘truncal’ mutations [8], we raised the read-depth threshold to focus on those variants with a position read-depth of ≥100 and looked for low frequency somatic variants that may have arisen recently in the clonal evolution process. The majority of variants found at a read-depth of ≥100 were already known and were excluded from analysis; however, novel variants were also detected that were unique to either the tumor or the node (Table 3). The lowest frequency unique variant detected by variant type (SNV, insertion, deletion, and substitution, respectively) was 0.88%, 3.7%, 10.07%, and 4.57% in the tumor, and 7.41%, 3.01%, 10.07%, 2.78% in the node). The SNV variant frequency increased from 0.88% to 7.41% from tumor to node, which could reflect sample differences, with a more heterogeneous mix of clones in the tumor, which then masks the presence of variants in the sample. The node; however, may represent a dominant clone that metastasized from the primary tumor but has only recently branched/evolved.

**Table 3:** Known and novel variant counts at a read-depth of ≥100 that overlapped genes and their flanking regions.

		Primary tumor	Axillary lymph-node	Unique to primary tumor	Unique to axillary lymph-node
**SNVs**	**Total calls**	57829	55203	1446	1400
	**dbSNP**	55823	53349	943	954
	**Not in dbSNP**	2006	1854	503	446
**Insertions**	**Total calls**	2721	2652	196	187
	**dbSNP**	2342	2296	73	71
	**Not in dbSNP**	379	356	123	116
**Deletions**	**Total calls**	2478	2431	93	98
	**dbSNP**	1972	1933	34	38
	**Not in dbSNP**	506	498	59	60
**Substitutions**	**Total calls**	1478	1503	239	222
	**dbSNP**	956	987	60	71
	**Not in dbSNP**	522	516	179	151

Variants were judged as known by their being listed in dbSNP. Variant counts for those unique to both samples are also shown. The lowest frequency variant detected for each variant type (SNV, insertion, deletion, and substitution, respectively) in each sample was 0.88%, 3.7%, 10.07%, and 4.57% in the tumor, and 7.41%, 3.01%, 10.07%, 2.78% in the node.

We also focused our analysis on variants called in the chromosomal instability 25 (CIN25) genes, shown to be predictive of poor clinical outcome in several cancers [34]. The majority of variants called in CIN25 genes were common to all samples (normal blood, tumor, and node), were called at comparable frequencies, and were already known and thus regarded as polymorphisms. We filtered out all variants called in the normal blood sample and thereafter found a single variant that was common to both tumor and node, as well as others that were unique to either the tumor or node (S1 Table). The majority of these variants were located upstream of the TSS in the region of RNA polymerase binding [35], which could result in altered expression of the target gene [36]–[38]. The single variant common to the tumor and node was an insertion upstream of the TSS of *TOP2A* at high frequency (86.1%, tumor; 82.4%, node), suggesting homozygosity in both or perhaps amplification of this locus. *TOP2A* is one of four genes, including *AURKA*, *FOXM1*, and *TPX2*, whose expression is significantly associated with tumor proliferation and can segregate breast cancers by outcome [39].

CIN25 variants unique to the node included a single base insertion (23.4% frequency) in the 3′ untranslated region (3′UTR) of *TGIF2*, and also a predominant three-base insertion (92.9% frequency) upstream of the TSS of the same gene. Given the variable frequencies of the two *TGIF2* variants, it suggests that at least two distinct clones predominate in the node, as suggested by Gerlinger [8]. TGIF2 is a DNA-binding homeobox and is a transcriptional repressor [40] that is highly expressed in ovarian cancer [41] and has been suggested as having an indirect role in metastasis through micro RNA methylation [42]. The only other variant unique to the node was an insertion upstream of the TSS of *CCNB2* (92.3% frequency), increased expression of which has been suggested to result in CIN in cancer [43].

## Conclusions

In conclusion, whole genome deep sequencing of a matched primary tumor and lymph node metastasis revealed largely overlapping alterations and that there were very few mutations unique to the involved node. Variants common to tumor and node include SNVs in *TP53* and *ARAP3*, which mediate rearrangements to the cytoskeleton and cell shape, and an insertion in *TOP2A*, whose expression is significantly associated with tumor proliferation and can segregate breast cancers by outcome. However, significant conclusions regarding early dissemination needs analysis of a larger cohort of samples.

### Sequence Data

All sequence data is available at the European Bioinformatics Institute (EBI) under accession number PRJEB7607 (ERP008528).

## Supporting Information

S1 Table **Variants in CIN25 genes: overlap between tumor and node.** Many variants were found in the region upstream of the TSS and could therefore alter the respective gene expression of each. Variants are described using the following syntax: variant type, base change, genomic position, gene region, functional impact, frequency.
(DOC)
