# Supplementary material for: Whole Genome Sequence Analysis Suggests Intratumoral Heterogeneity in Dissemination of Breast Cancer to Lymph Nodes
Source: PLoS One. 2014 Dec 29;9(12):e115346. doi: 10.1371/journal.pone.0115346 (PMC4278903; doi:10.1371/journal.pone.0115346)
Supplement: S1 Table — Variants in CIN25 genes: overlap between tumor and node. Many variants were found in the region upstream of the TSS and could therefore alter the respective gene expression of each. Variants are described using the following syntax: variant type, base change, genomic position, gene region, functional impact, frequency. (DOC) [file pone.0115346.s001.doc]

| **CIN25 gene [34]** | **Description and role [34]** | **Primary tumor** | **Axillary lymph-node** |
| --- | --- | --- | --- |
| *TPX2* | Is required for normal spindle morphology and centrosome integrity. Its overexpression provokes polyploidisation. It is required for targeting Aurora-A kinase to the spindle apparatus. | NA | NA |
| *PRC1* | Critical to the formation of the central spindle. Contributes to the correct formation of the spindle during the metaphase. | Ins, CTCA, 91542216, TSS-upstream, unknown, 12.2% | NA |
| *FOXM1* | Is a key transcriptional regulator of genes involved in chromosomal segregation and cytokinesis. | NA | NA |
| *CDC2* | Play important regulatory roles in cell cycle control. Plays an important role in the proper timing of mitotic spindle formation. | NA | NA |
| *TGIF2* | - | NA | Ins, AAA, 35199658, TSS-upstream, unknown, 92.9% |
|  |  |  | Ins, T, 35220788, 3`UTR, unknown, 23.4% |
| *MCM2* | Involved in the initiation of eukaryotic genome replication. | NA | NA |
| *H2AFZ* | This particular histone is required for embryonic development. | NA | NA |
| *TOP2A* | Plays an essential catalytic role in chromosome segregation. | Ins, T, 38575393, TSS-upstream, unknown, 86.1% | Ins, T, 38575393, TSS-upstream, unknown, 82.4% |
| *PCNA* | Is involved in the RAD6-dependent DNA repair pathway. | NA | NA |
| *UBE2C* | Is required for the destruction of mitotic cyclins. | NA | NA |
| *MELK* | Is a potential regulator of the G2/M progression and may act antagonistically to the CDC25B phosphatase. | NA | NA |
| *TRIP13* | - | NA | NA |
| *CNAP1* | Is responsible for targeting CNAP1, and possibly condensin, to mitotic chromosomes. | NA | NA |
| *MCM7* | Involved in the initiation of eukaryotic genome replication. | NA | NA |
| *RNASEH2A* | Shows increased activity during DNA replication. | NA | NA |
| *RAD51AP1* | - | NA | NA |
| *KIF20A* | Is required for cytokinesis. Relocation of the Aurora B/INCENP/survivin passenger protein complex requires KIF20A | NA | NA |
| *CDC45L* | Required to the initiation of DNA replication | NA | NA |
| *MAD2L1* | Is a component of the mitotic spindle assembly checkpoint that prevents the onset of anaphase until all chromosomes are properly aligned at the metaphase plate p31(comet, formerly known as Cmt2) counteracts the function of Mad2 and is required for the silencing of the spindle checkpoint. Reconstitution of Mad2 in Brca1deficient cells partially restored the spindle checkpoint. Binds directly to CDC20. | NA | NA |
| *ESPL1* | Is required for sister chromatid separation during mitosis in human cells. | Sub, TG>GT, 53659217-9, TSS-upstream, unknown, 94.7% | NA |
|  |  | SNV, C>G, 53659480, TSS-upstream, unknown, 31.4% |  |
| *CCNB2* | Is a member of the cyclin family and its overexpression results in CIN. | Ins, TT, 59396818, TSS-upstream, unknown, 75% | Ins, T, 59396818, TSS-upstream, unknown, 92.3% |
| *FEN1* | Causes repeat instability and aberrant DNA repair. | NA | NA |
| *TTK* | Is required for centrosome duplication and for the normal progression of mitosis. Is a critical regulator of genetic stability, it is required for the spindle assembly checkpoint. | SNV, G>T, 80709554, TSS-upstream, unknown, 76.9% | NA |
|  |  | SNV, C>T, 80710492, TSS-upstream, unknown, 84.9% |  |
|  |  | SNV, C>A, 80712497, TSS-upstream, unknown, 78.6% |  |
| *CCT5* | - | NA | NA |
| *RFT4* | Mutation in this replication factor leads to aberrant checkpoint control in response to damage to chromosomes or DNA damage. | NA | NA |
